# Effect of Colloid Centrifugation and Cold Storage of Boar Semen at 4 °C on Bacterial Load and Sperm Quality

**DOI:** 10.3390/antibiotics14030267

**Published:** 2025-03-06

**Authors:** CongBang Ngo, Jane M. Morrell, Ingrid Hansson, Padet Tummaruk, Aleksandar Cojkic

**Affiliations:** 1 Clinical Sciences, Swedish University of Agricultural Sciences (SLU), 756 51 Uppsala, Sweden; congbangdhnl39@gmail.com (C.N.); aleksandar.cojkic@slu.se (A.C.); 2 Department of Obstetrics, Gynaecology and Reproduction, Faculty of Veterinary Science, Chulalongkorn University, Bangkok 10330, Thailand; padet.t@chula.ac.th; 3 Animal Biosciences, Swedish University of Agricultural Sciences (SLU), P.O. Box 7054, 750 07 Uppsala, Sweden; ingrid.hansson@slu.se

**Keywords:** hypothermic semen storage, alternatives to antibiotics, Porcicoll

## Abstract

**Background/Objectives**: Alternatives to antibiotics in semen extenders used for pig breeding are needed. Cold storage of boar semen is possible in certain extenders; however, bacteria can still survive. Colloid centrifugation can remove bacteria; therefore, a combination of colloid centrifugation and cold storage on the bacterial load and sperm quality of boar semen was investigated. **Results**: The sperm quality in the original samples were similar in samples with and without antibiotics, respectively. At Day 4, the bacterial count was not different between controls stored at 17 °C with antibiotics and SLC cold-stored samples without antibiotics. The proportion of dead sperm increased in controls at 16–18 °C (28.12 ± 21.72% vs. 32.88 ± 20.94%; *p* < 0.05), and DNA fragmentation increased from 1.5 ± 1.02% at Day 0 to 6.1 ± 3.64% at Day 4 (*p* < 0.01), whereas it did not deteriorate in the cold-stored samples (Day 4: %DFI 2.00 ± 1.65% and 1.90 ± 1.05% for SLC samples with and without antibiotics, respectively). **Methods**: Aliquots of boar ejaculates were extended in Androstar Premium with and without antibiotics. The samples without antibiotics were processed by Single Layer Centrifugation (SLC) through Porcicoll; sperm pellets were resuspended in AndroStar Premium, with or without antibiotics, cooled slowly, and stored for 4 days at 4 °C. Controls (aliquots extended in AndroStar Premium with antibiotics) were stored at 17 °C. The bacterial load and sperm quality were assessed in the original samples and after 4 days of storage. **Conclusions**: Single Layer Centrifugation combined with cold storage is an effective method for reducing the bacterial load in boar semen, with or without antibiotics.

## 1. Introduction

Antimicrobial resistance is a global problem necessitating novel alternatives to the use of antibiotics [1]. One non-therapeutic use of antibiotics is in semen extenders used when preparing semen doses for artificial insemination, which is a common method of breeding production animals and horses [2]. The mucosa of the reproductive tract has a microbiota [3,4] that serves as a protective barrier against pathogens [5]. Some of these bacteria [6], and bacteria from other sources [7], are transferred to the ejaculate during semen collection; additional bacteria may contaminate the ejaculate during preparation and processing of semen doses [8,9]. Since boar spermatozoa are particularly sensitive to cooling, boar semen doses for insemination are usually transported and stored at 16–18 °C [10]. However, this temperature permits the growth of some bacteria, facilitated by the nutrient medium provided by the semen extender [2]. These bacteria can have a deleterious effect on the sperm quality during storage [11] and may cause disease in inseminated sows. Therefore, antibiotics are added to semen extenders, according to national and international regulations, e.g., [12].

Pig artificial insemination uses large volumes of liquid semen (typically 80–90 mL per dose), which leaves the reproductive tract by backflow [13], exposing bacteria in the environment to the antibiotics. There are some reports of antimicrobial resistance (AMR) occurring in the bacteria found in boar semen [14,15,16]. It is even possible for resistance genes to be transferred from dead bacteria [17]. Thus, the expelled liquid after artificial insemination (AI) represents a considerable opportunity to increase AMR in environmental bacteria or even in bacteria in the sows themselves or in barn personnel, making this a One Health issue.

Alternatives to antibiotics in semen extenders are being investigated. Several possibilities were presented in a recent review [18], including cold storage, freezing semen, using substances that have antimicrobial activity that are not used therapeutically, and physical separation of spermatozoa from seminal plasma and bacteria, especially centrifugation through a single layer of colloid (SLC). Recently, some commercial extenders were developed that can be used for cold storage of boar semen, such as AndroStar Premium, which can be used for storage of boar semen at either 16–18 °C or for cold storage [19,20]. Sperm motility and membrane integrity were not adversely affected by cold storage; however, the bacterial load may increase during storage even in samples stored at 5 °C [20]. Depending on the species, these bacteria could potentially be a threat to sow health and reproductive efficiency. Some insemination trials were carried out on semen that had been prepared in antibiotic-free extenders and cooled, but antibiotics were added before insemination. Therefore, no information is available regarding the bacterial load of these insemination doses after the addition of antibiotics [19].

One possible solution to the presence of bacteria would be to reduce the bacterial load by physical means. The ability of centrifugation through a high-density colloid to remove bacterial contamination from boar semen was shown previously [21], but some of the spermatozoa were lost as they become caught at the semen/colloid interface, together with dead or damaged spermatozoa. Further studies scaled-up the technique to allow processing of whole ejaculates [22], while other studies showed that lower-density colloids could be used to optimize sperm retrieval without having a detrimental effect on the sperm quality [23,24]. The sperm quality was maintained in the SLC samples during storage at 16–18 °C [23]. Low-density Porcicoll was subsequently used to prepare boar semen for AI trials, resulting in the birth of offspring [18].

A field trial separating spermatozoa from seminal plasma and bacteria by SLC through a low-density colloid was reported recently [18,25]. In the first of these studies, a small number of sows was inseminated with boar sperm prepared by centrifugation through low-density Porcicoll. Pregnancy rates, farrowing rates, and litter size were not different from controls, and fewer mummified fetuses were seen in the litters in the SLC group [18]. In the second SLC study, the semen was prepared on the farm using low-density Porcicoll, and the resulting sperm preparations were used for insemination. Farrowing rates and litter sizes were not different between sows inseminated with SLC-prepared sperm samples and those inseminated with control sperm doses (conventional semen doses with gentamicin); however, the number of piglets born dead was less in the SLC group [25]. These results with SLC are encouraging but it is not known whether SLC-selected boar spermatozoa can withstand cold storage. Furthermore, there are no published data available on the number of bacteria in the SLC-sperm doses when the semen doses are stored cold. Therefore, in the present study, ejaculates were split; one portion of each ejaculate was stored conventionally, the other was prepared by SLC, cooled slowly [26], and subjected to cold storage at 4 °C for 4 days. The bacterial load and sperm quality in cold-stored SLC and conventionally prepared semen doses from the same ejaculates were compared.

## 2. Results

### 2.1. Bacterial Load

**Figure 1:**
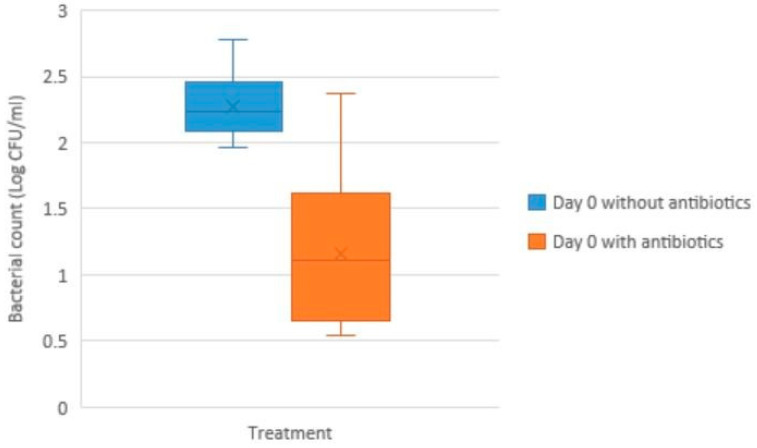
**Bacterial load in boar semen samples (log colony forming units/mL, determined by plate count agar) on arrival at the laboratory (Day 0), with or without antibiotics in the semen extender (n = 9 in each group).** Note: The box represents the interquartile range (IQR), which spans from the first quartile (Q1) to the third quartile (Q3). The median (the horizontal line inside the box) divides the dataset into two equal halves. The whiskers (the vertical lines above and below the box) show the main range of the data, extending up to 1.5 times the IQR from Q1 and Q3. Any outliers appear as individual points beyond the whiskers. X represents the mean (average) of the data. There was no difference in bacterial count between the two groups.

On arrival at SLU, the mean bacterial load was 1.2 log CFU/mL in the samples with antibiotics compared to 2.4 log CFU/mL in samples without antibiotics (Figure 1). These bacterial counts are not significantly different (*p* > 0.05).

### 2.2. Sperm Quality

On arrival at the laboratory (Day 0), the sperm quality in the samples did not differ between samples with and without antibiotics (Table 1).

### 2.3. Effect of Single Layer Centrifugation

**Figure 2:**
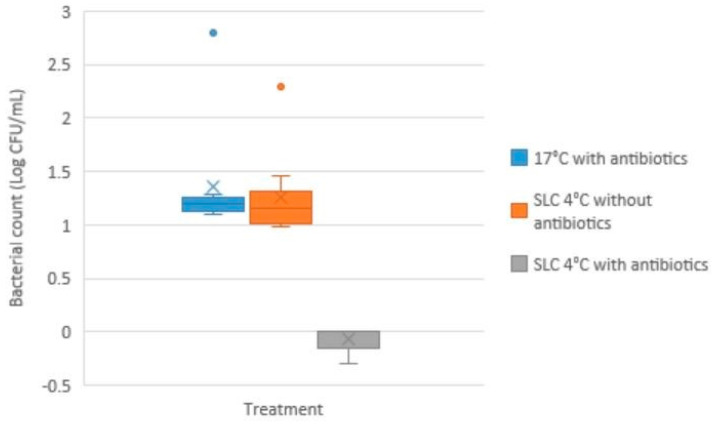
**Bacterial load as log colony forming units/mL (CFU/mL), determined by plate count agar, in boar semen samples after 4 days of storage, either as conventional doses stored at 16–18 °C, or cold-stored 4 °C after Single Layer Centrifugation (n = 9 in each group).** Note: The box represents the interquartile range (IQR), which spans from the first quartile (Q1) to the third quartile (Q3). The median (the horizontal line inside the box) divides the dataset into two equal halves. The whiskers (the vertical lines above and below the box) show the main range of the data, extending up to 1.5 times the IQR from Q1 and Q3. Any outliers would appear as individual points beyond the whiskers. X represents the mean (average) of the data. There were no differences in bacterial counts between treatments.

The yield after SLC was 84 ± 15%. After 4 days of conventional storage (16–18 °C) in the presence of antibiotics, the bacterial load of the controls, as determined by plate count agar, was similar to the SLC cold-stored samples (NS; *p* > 0.05) (Figure 2).

There were some differences in the sperm quality in the controls after storage for 4 days (Table 2), for example, an increase in the proportion of dead sperm from 28.12 ± 7.2% to 32.88 ± 20–94% (*p* < 0.05). Notably, there was a significant increase in %DFI, from 1.5 ± 1.02% on Day 0 to 6.1 ± 3.64% on Day 4 (*p* < 0.01). However, there were no differences in the sperm quality between the controls stored at 16–18 °C for 4 days and the cold-stored SLC samples, with the exception of DNA fragmentation, which was significantly less in the SLC cold-stored samples than in the stored controls (*p* < 0.05; Table 2).

## 3. Discussion

The purpose of this study was to compare the bacterial load and sperm quality in boar sperm samples in extenders with or without antibiotics stored at 4 °C for 4 days after SLC, with conventionally prepared samples, i.e., in extenders with antibiotics (controls) stored at 16–18 °C for the same period. The results showed that bacteria could be cultured from the conventional semen samples, even after 4 days of storage at 16–18 °C in the presence of antibiotics. However, the sperm quality was not adversely affected by the combination of SLC and cold storage, and some properties, such as chromatin integrity, were better in the cold-stored samples than in the conventionally stored samples. Bacterial counts (CFU/mL) between SLC samples and controls were not different; however, this may have been due to the small sample size. Further investigation with a larger sample size and additional breeds of boar is warranted.

Our results are somewhat different from those of Menezes et al. [20], who showed an increased bacterial load during storage at 17 °C; however, they found that extended semen without antibiotics had a greater bacterial content than semen with antibiotics. This difference could be due to differences between animals or farms, or the hygienic measures during semen collection. What was striking, however, was that the bacterial load in our cold-stored SLC samples without antibiotics did not have a greater bacterial load than the control samples with antibiotics, indicating that this method could be employed to advantage to reduce usage of antibiotics in pig breeding.

These results are similar to previous studies on the bacterial load in boar semen samples, where the bacterial count increased in controls during storage, whereas it remained the same or declined slightly in samples stored at 5 °C [19]. However, whether or not bacteria can survive during cold storage appears to depend on the particular bacterial species present, since there are reports of increased growth of bacteria that are known to have a negative impact on fertility, e.g., *Serratia marcescens*, even during cold storage [20]. However, in another study, no increased growth of either *Serratia marcescens* or *Klebsiella oxytoca* was reported after cold storage, whereas these bacteria apparently did grow at 17 °C [27,28,29]. Our present results on reduction in bacterial numbers are in agreement with those of Lacalle et al. [11], who showed that SLC through a low-density colloid not only drastically reduced the bacterial count but, furthermore, effectively removed *Burkholderia ambifaria* and *Staphylococcus simulans* from spiked boar semen samples and considerably reduce counts of *Pseudomonas aeruginosa* [11].

The maintenance of sperm DNA integrity during cold storage is interesting and confirms earlier observations in our laboratory. In previous studies, an increase in DNA fragmentation (%DFI) was seen during storage at 17 °C [30], which was not seen during cold storage [26]. These results were also similar to those reported by Waberski et al. for cold storage but there was no increase in DNA fragmentation in their controls stored at 17 °C [19]. It is possible that there is a breed or male effect, resulting in an increase in DNA fragmentation in some semen samples during storage at 17 °C, but in any case, the DNA damage is apparently circumvented by cold storage, as shown again here. The semen used in the present study came from Hampshire boars, as did the semen used in our previous study [26]. This breed is popular as a terminal sire, and therefore the semen is readily available here in Sweden. However, in a different study where there was also an increase in %DFI during storage, the semen originated from Norwegian Landrace boars [30]. In a different study [19], where no increase in sperm DNA fragmentation was observed during storage, the semen came from Piétrain, Duroc, German Landrace and Large White boars. Thus, there may be a breed difference in the susceptibility of sperm DNA to increased fragmentation during conventional storage, which should be investigated in the future.

The cooling procedure used in the present experiment (first cooling to 17–23 °C for a period of approximately 6 h during transport from the boar stud and manipulations in the laboratory, then cooling from room temperature down to 4 °C over approximately 6 h), is faster than the controlled cooling rate used by Luther et al. [27,28]. However, it represents a practical method for slowly reducing the temperature of boar sperm samples without affecting their fertilizing ability [26]. As such, it offers the possibility for breeding companies to cool boar semen without investing in elaborate cooling equipment. An AI trial is currently underway, using a combination of SLC and cold storage, to assess the feasibility for the production of commercial boar semen doses.

## 4. Materials and Methods

### 4.1. Semen Samples

Ejaculates were obtained from each of 9 Hampshire boars (one ejaculate per boar) at a commercial supplier (Köttforetagen, Hållsta, Sweden). The animals were kept and handled according to national and international regulations on the housing and care of animals. Ejaculates that met the company’s criteria for commercial semen doses, i.e., >70% motility and >70% normal morphology (with less than 20% of a specific defect), were used in this study to prepare semen doses containing approximately 2 × 10^9^ spermatozoa in an 80 mL extender. One standard insemination dose from each boar was prepared in AndroStar Premium with antibiotics while the other dose was extended in AndroStar Premium without antibiotics (kind gift of Minitube International, Tiefenbach, Germany).

### 4.2. Experimental Design

**Figure 3:**
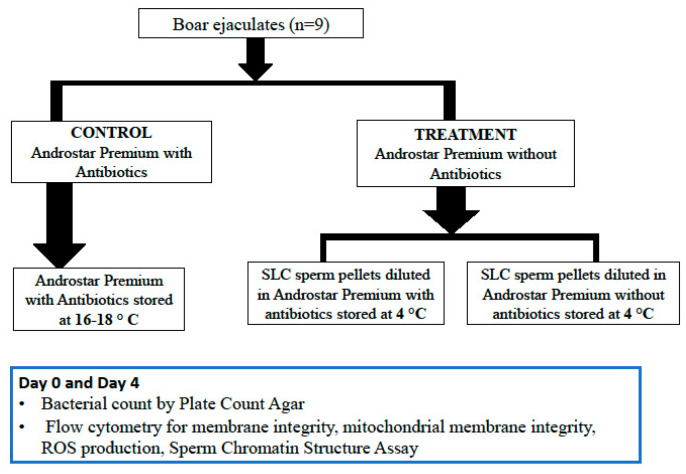
**Experimental design.** Note: SLC = Single Layer Centrifugation. Note: n = 9 in each group.

The experimental design is shown in Figure 3.

### 4.3. Sample Preparation

The semen doses were transported to the laboratory at the Swedish University of Agricultural Sciences in an insulated box, arriving approximately 4 h after semen collection. At the laboratory, the sperm concentration of each sample was measured using a Nucleocounter SP-100, according to the instructions for use. One aliquot of each sample was taken immediately for flow cytometry; a second aliquot was placed in the refrigerator for subsequent bacterial count using plate count agar. The samples in AndroStar Premium without antibiotics were used for SLC. The samples in AndroStar Premium with antibiotics were stored in a climate-controlled box at 16–18 °C as controls.

### 4.4. Single Layer Centrifugation

An aliquot (15 mL) of low-density Porcicoll was poured into a 50 mL centrifuge tube; the semen sample (20 mL) was carefully pipetted on top, avoiding mixing [15]. The tube was centrifuged at 300 g for 20 min, after which the supernatant was removed with a water pump, and the sperm pellet was resuspended in AndroStar Premium, either with or without antibiotics. Thus, there were two SLC preparations per boar, one with antibiotics in the semen extender and the other without antibiotics. The sperm concentration was measured again to allow the yield to be calculated. These SLC preparations were placed in an insulated box, inside another insulated box, in the cold room at 4 °C [26] approximately 2 h after arrival at the laboratory. This allows the samples to cool to 4 °C in approximately 6 h. Four days later, aliquots of the cold-stored SLC samples and the controls were evaluated for sperm quality and for bacterial load.

### 4.5. Bacterial Load

The sperm samples were cultured on plate count agar under aerobic conditions to determine the number of viable bacteria, according to the standard protocol (NMKL 86, 5 Ed., 2013). After diluting 1 mL of each sperm sample 1:10 with peptone diluent (Dilucup^®^ Elegance MRD; LabRobot Products AB, Stenungsund, Sweden), the samples were homogenized by shaking on a Dilugent^®^ Shaker (LabRobot Products AB, Sweden). The diluted samples (1.0 mL aliquots) were pipetted into 9 cm diameter Petri dishes in duplicate; 10–15 mL plate count agar (PCA; Oxoid, Basingstoke, UK) was poured into each dish. After agitating the Petri dishes gently to distribute the bacteria evenly, the agar was allowed to solidify. Subsequently, an additional 10–15 mL of PCA was added to each dish and allowed to solidify. The plates were then incubated for 72 h at 30 °C. The number of colony forming units (CFU) was enumerated using a colony counter (Gerber Instruments, Im Langhag, Switzerland) and calculated from the standard formula: N = ∑C/(V × 1.1 × d), where N = number of micro-organisms, ∑C = sum of colonies on the two plates from successive dilutions, V = volume of the inoculum/plate (mL), and d = 1st countable dilution retained [31].

### 4.6. Flow Cytometry

Sperm quality was assessed by flow cytometry as described by Hallberg et al. [26] using a FACSVerse flow cytometer (BDBiosciences; Franklin Lakes, NJ, USA) equipped with standard optics. Sperm samples were diluted to 2 × 10^6^ spermatozoa/mL with modified Beltsville Thawing Solution (BTS; Minitube International, Tiefenbach, Germany).

#### 4.6.1. Membrane Integrity

Diluted sperm samples were stained with 0.6 µL SYBR-14 (1:50 in BTS; 40 nM) and 3 µL propidium iodide (PI; 24 μM) (Live-Dead Sperm Viability Kit L-7011; Invitrogen, Eugene, OR, USA). The tubes with 300 µL aliquots were incubated in the dark at 38 °C for 10 min. Excitation was made with a blue laser (emitting at 488 nm); green fluorescence (FL1) from SYBR-14 red fluorescence (FL3) from PI were detected with band-pass filters (527/32 nm and 700/54 nm, respectively). Measurements from 50,000 events were collected and quantified as proportions (%) in the following categories: live (SYBR14+, PI−), dead (SYBR−, PI+), or dying (SYBR14+, PI+).

#### 4.6.2. Reactive Oxygen Species

Aliquots (300 µL) of all sperm samples were stained with 3 µL of Hoechst 33258 (HO; Sigma, Stockholm) at a concentration of 40 mM solution, 3 µL of 40 mM hydroethidine (HE; Invitrogen Molecular Probes, Eugene, OR, USA), and 3 µL of 2 mM dichlorodihydrofluorescein diacetate (DCFDA; Invitrogen Molecular Probes). After incubating the samples in the dark at 37 °C for 30 min, they were analyzed by flow cytometry. Excitation was from a blue laser and a violet laser emitting at 488 nm and 405 nm, respectively. Fluorescence from DCFDA (green) was detected with a band-pass filter FL1 (527/32 nm), red fluorescence from HE was detected with a band-pass filter FL3 (700/54 nm), and fluorescence from Hoechst 33258 (blue/green) was detected via a band-pass filter FL5 (528/45 nm). In total, 30,000 sperm-specific events were evaluated per sample. On the FSC-SSC dotplot, debris was gated out, and the proportions of spermatozoa were classified as living or dead superoxide or hydrogen peroxide negative, and living or dead superoxide or hydrogen peroxide positive (%).

#### 4.6.3. Mitochondrial Membrane Potential

The sperm samples (300 µL aliquots) were stained with 1.2 µL of 3 mM JC-1 (Molecular Probes), which is the lipophilic cationic probe 5,5′,6,6′-tetrachloro-1,1′,3,3′-tetraethylbenzimidazolyl-carbocyanine iodide. After incubating the mixture in the dark at 37 °C for 30 min, fluorescence was evaluated by flow cytometry. Excitation was obtained with a blue laser (488 nm); emitted fluorescence was detected using both FL1 (527/32 nm) and FL2 (586/42 nm) filters, applying compensation between channels. For each sample, sperm-specific events were evaluated. Using the dotplot, debris was gated out and 30,000 spermatozoa were classified according to their fluorescence, as spermatozoa with high MMP (%, orange fluorescence) and those with low MMP (%, green fluorescence).

#### 4.6.4. Sperm Chromatin Structure Assay

Aliquots (50 µL) of each sperm sample were diluted 1:1 with TNE buffer (Tris-sodium chloride-EDTA; containing 0.15 mol/L NaCl, 0.01 mol/L Tris-HCl, 1 mmol/L EDTA, at pH 7.4). All diluted samples were snap-frozen in liquid nitrogen and were stored in a −80 °C freezer until analysis. Then they were thawed on crushed ice, and aliquots (10 μL) were mixed with TNE buffer (90 μL) before adding 200 µL of a solution containing 0.17% Triton X-100, 0.15 mol/L NaCl, and 0.08 mol/L HCl, at pH 1.2. Acridine orange stain (600 µL) (6 μg/mL in 0.1 mol/L citric acid, 0.2 mol/L Na_2_HPO_4_, 1 mmol/L EDTA, 0.15 mol/L NaCl, pH 6.0) was added at room temperature. The samples were analyzed by flow cytometry within 3–5 min of adding the acridine orange. At least 10,000 events were analyzed for each sample, at 200 cells/s, after excitation with a blue laser (488 nm). Forward scatter (FSC) and side scatter (SSC) were collected for analysis, FL1 green fluorescence (FL1) was collected through a band-pass filter (527/32 nm), and red fluorescence (FL3) was collected using a band-pass filter (wavelengths 700/54 nm). Spermatozoa were gated in the FSC-SCC dotplot. An index of the ratio of spermatozoa with red fluorescence (single stranded DNA) to red and green fluorescence (single and double-stranded DNA) was calculated (DNA fragmentation index; %DFI) using Flow Cytometry Standard (FCS) Express software, version 5 (De Novo Software, Pasadena, CA, USA).

### 4.7. Statistical Analysis

All statistical analyses were performed using R version 4.4.2. Linear mixed-effects models were conducted to analyze the data, with fixed effects for treatment and antibiotics and a random effect for ejaculates. Subsequently, post-hoc pairwise comparisons were conducted using *t*-tests to evaluate the differences between groups. The significance threshold for all tests was set with three levels of significance (*p* < 0.05, *p* < 0.01, and *p* < 0.001). Data are presented as mean ± standard deviation (SD).

## 5. Conclusions

Bacteria were cultured from conventional semen samples containing antibiotics after 4 days of storage at 16–18 °C, and from SLC samples cooled and stored at 4 °C for 4 days, and the sperm quality was evaluated. There was no difference in the bacterial load and no adverse effect on the sperm quality when boar semen was prepared by Single Layer Centrifugation and stored for 4 days at 4 °C without antibiotics compared to conventional storage with antibiotics at 16–18 °C. Therefore, it might be possible to use this method as an alternative to conventional storage, with the advantage that it might be easier to maintain the correct temperature during transport and storage at 4 °C than at 16–18 °C. The possibility of avoiding adding antibiotics to the semen extender would be of considerable benefit from a One Health perspective.

## Figures and Tables

**Table 1:** Sperm quality (mitochondrial membrane potential, membrane integrity, and DNA fragmentation; mean ± SD) analyzed by flow cytometry in boar sperm samples on arrival at the laboratory (Day 0) in extender with and without antibiotics (n = 9).

Parameter (%)	Semen in Extender with Antibiotics	Semen in Extender Without Antibiotics
MMP Low	28.80 ± 9.50	27.04 ± 8.61
MMP High	71.20 ± 9.50	72.96 ± 8.61
MI Living	56.66 ± 19.97	50.94 ± 11.63
MI Dead	28.12 ± 21.77 ^a^	35.82 ± 14.76 ^a^
MI Dying	15.22 ± 5.07	13.24 ± 4.80
Dead H_2_O_2_ −	34.21 ± 6.48	36.26 ± 7.05
Dead H_2_O_2_ +	0.98 ± 1.30	0.30 ± 0.17
Live H_2_O_2_ −	58.87 ± 9.40	58.89 ± 10.26
Live H_2_O_2_ +	5.94 ± 6.32	4.55 ± 4.80
Live SO −	31.73 ± 15.77	31.34 ± 13.20
Live SO +	30.96 ± 18.53	30.01 ± 13.41
Dead SO +	34.28 ± 6.90	35.04 ± 6.16
%DFI	1.51 ± 1.02	1.47 ± 0.67

Notes: MMP = mitochondrial membrane potential; MI = membrane integrity; H_2_O_2_ = hydrogen peroxide, SO = superoxide; %DFI = DNA fragmentation index. ^a^: *p* < 0.05.

**Table 2:** Sperm quality (mitochondrial membrane potential, membrane integrity, and DNA fragmentation; mean ± SD) analyzed by flow cytometry) in boar sperm samples stored for 4 days at either 16–18 °C in the presence of antibiotics (controls) or cold-stored (4 °C) after Single Layer Centrifugation, with or without antibiotics (n = 9).

Sperm Parameters (%)	Control Semen with Antibiotics, 16–18 °C Day 4	SLC Samples with Antibiotics, 4 °C Day 4	SLC Samples Without Antibiotics, 4 °C Day 4
MMP Low	23.72 ± 11.74	14.80 ± 8.14	15.70 ± 12.17
MMP High	76.28 ± 11.74	85.20 ± 8.14	84.30 ± 12.17
MI Living	52.03 ± 21.24	50.03 ± 14.65	52.54 ± 14.03
MI Dead	32.88 ± 20.94	28.65 ± 15.63	36.23 ± 15.02
MI Dying	15.09 ± 4.89	11.32 ± 6.06	11.23 ± 5.53
Dead H_2_O_2_ −	35.83 ± 24.6	38.37 ± 15.99	37.83 ± 14.85
Dead H_2_O_2_ +	15.74 ± 20.45	9.64 ± 9.85	7.58 ± 5.55
Live H_2_O_2_ −	35.42 ± 18.4	44.95 ± 13.52	45.73 ± 13.53
Live H_2_O_2_ +	13.05 ± 15.09	7.03 ± 4.28	8.86 ± 4.94
Live SO −	26.09 ± 12.74	25.93 ± 11.69	25.19 ± 11.53
Live SO +	19.24 ± 16.03	28.65 ± 9.04	30.22 ± 7.25
Dead SO +	48.41 ± 14.34	41.65 ± 9.31	40.60 ± 11.17
%DFI	6.11 ± 3.64 ^ab^	2.00 ± 0.65 ^a^	1.90 ± 1.05 ^b^

Notes: MMP = mitochondrial membrane potential; %DFI = DNA fragmentation index; SLC = Single Layer Centrifugation; H_2_O_2_ = hydrogen peroxide, SO = superoxide; same superscript within a column indicates statistical significance at ^a^ *p* < 0.05, ^b^ *p* < 0.001.

## Data Availability

All data are presented in the manuscript.
